# Risk factors for COVID-19 case fatality rate in people with type 1 and type 2 diabetes mellitus: A nationwide retrospective cohort study of 235,248 patients in the Russian Federation

**DOI:** 10.3389/fendo.2022.909874

**Published:** 2022-08-09

**Authors:** M. V. Shestakova, O. K. Vikulova, A. R. Elfimova, A. A. Deviatkin, I. I. Dedov, N. G. Mokrysheva

**Affiliations:** Endocrinology Research Centre, Moscow, Russia

**Keywords:** diabetes mellitus, COVID-19, case fatality rate, anti-COVID-19 vaccination, glucose-lowering therapy

## Abstract

**The aim:**

To study the association of demographic, clinical, and laboratory factors and the use of glucose-lowering drugs and anti-coronavirus disease (COVID-19) vaccination with the COVID-19-related case fatality rate (CFR) in diabetes mellitus (DM) patients.

**Methods:**

This study is a nationwide observational cohort study based on the data from the National Diabetes Register (NDR) that is the database containing online clinical information about the population with DM. The outcomes (death or recovery) for COVID-19 were registered in 235,248 patients with DM [type 1 diabetes mellitus (T1DM), n = 11,058; type 2 diabetes mellitus (T2DM), n = 224,190] from March 20, 2020, until November 25, 2021. The unadjusted odds ratio (OR) and 95% confidence interval (CI) were used to estimate the risk factors for CFR. Then the ranging of significant factors was performed and the most vulnerable groups of factors for the lethal outcome were chosen.

**Results:**

The CFR due to COVID-19 was 8.1% in T1DM and 15.3% in T2DM. Increased CFR was associated with the male population [OR = 1.25 (95% CI: 1.09–1.44) in T1DM and 1.18 (95% CI: 1.15–1.21) in T2DM], age ≥65 years [OR = 4.44 (95% CI: 3.75–5.24) in T1DM and 3.18 (95% CI: 3.09–3.26) in T2DM], DM duration ≥10 years [OR = 2.46 (95% CI: 2.06–2.95) in T1DM and 2.11 (95% CI: 2.06–2.16) in T2DM], body mass index (BMI) ≥30 kg/m^2^ [OR = 1.95 (95% CI: 1.52–2.50)] in T1DM, HbA1c ≥7% [OR = 1.35 (95% CI: 1.29–1.43)] in T2DM. The atherosclerotic cardiovascular disease (ASCVD) and chronic kidney disease (CKD) were associated with higher CFR in T1DM but not in T2DM. The pre-COVID-19 glucose-lowering therapy in T2DM was differently associated with CFR (OR): 0.61 (95% CI: 0.59–0.62) for metformin, 0.59 (95% CI: 0.57–0.61) for dipeptidyl peptidase-4 inhibitors (DPP-4 inhibitors), 0.46 (95% CI: 0.44–0.49) for sodium-glucose co-transporter-2 (SGLT2) inhibitors, 0.38 (95% CI: 0.29–0.51) for glucagon-like peptide-1 receptor agonists (arGLP-1), 1.34 (95% CI: 1.31–1.37) for sulfonylurea (SU), and 1.47 (95% CI: 1.43–1.51) for insulin. Anti-COVID-19 vaccination was associated with a lower fatality risk in both DM types: OR = 0.07 (95% CI: 0.03–0.20) in T1DM and OR = 0.19 (95% CI: 0.17–0.22) in T2DM.

**Conclusions:**

The results of our study suggest that increased COVID-19-related fatality risk in both T1DM and T2DM patients associated with the male population, older age, longer DM duration, and absence of anti-COVID-19 vaccination. In T2DM, pre-COVID-19 glucose-lowering therapy with metformin, DPP-4 inhibitors, SGLT2 inhibitors, and arGLP-1 had a positive effect on the risk of death. The most vulnerable combination of risk factors for lethal outcome in both DM types was vaccine absence + age ≥65 years + DM duration ≥10 years.

## Introduction

The first coronavirus disease 2019 (COVID-19) cases occurred in December 2019 (1). Several months later, the infection spread globally, affecting almost every area of human life. In total, as of March 20, 2022, WHO published data of over 6 million registered deaths caused by COVID-19 and over 468 million confirmed cases (2). According to the nationwide statistics, as of May 20, 2022, 18.28 million COVID-19 cases were registered and 378,000 are deceased (3) in Russia. In other words, the case fatality rate (CFR) was near 2.1%. The real burden of the pandemic could be even higher; a comparison of excessive mortality and COVID-19-related death numbers suggested that infection impact is significantly underestimated (4). The coronavirus pandemic has negatively impacted the management of diabetes mellitus (DM) patients (5). The mortality risk for COVID-19 is not the same for different population cohorts (6). Infection of diabetic patients by Severe acute respiratory syndrome-related coronavirus 2 (SARS-CoV-2) results in a more severe course of COVID-19 and increases the risk of complications and poor outcomes. The male population, older age, and worse glycemic control associated with increased COVID-19 mortality in people with diabetes (7–10). At the same time, it remains controversial which kind of antidiabetic therapy should be continued or canceled in infected patients (11). It was demonstrated that COVID-19 mortality is higher in patients taking insulin and lower in patients taking metformin (12). At the same time, the effect of drug intake depends on the severity of coronaviral infection. For example, the use of metformin and sodium-glucose co-transporter-2 (SGLT2) inhibitors in critically ill patients is not recommended (13, 14). However, these results should be taken with caution due to the absence of a clear indication to change the therapy of diabetic patients with coronavirus infection. Additional large-scale studies are necessary to clarify the situation. Still, there is a lack of information concerning the preventive effects of anti-COVID-19 vaccination on the mortality rate of patients with diabetes.

The comprehensive evaluation of the course outcomes in the representative dataset of the affected population could reveal risk factors and therapy associated with increased mortality. In our study, we analyzed the data of more than 235,000 patients with DM of the National Diabetes Register (NDR) with a reported outcome of coronavirus infection.

The aim of the study was to estimate the CFR and risk factors for death in people with type 1 diabetes mellitus (T1DM) and type 2 diabetes mellitus (T2DM) with confirmed or highly suspected COVID-19.

## Materials and methods

### Study design and data sources

We performed a nationwide observational cohort study based on the data from the NDR to assess the risk factors associated with COVID-19-related death. The clinical and epidemiological monitoring of DM in the Russian Federation was performed through the NDR since 1996. The methodological and organizational reference center for NDR is the Endocrinology Research Centre (Moscow, Russia). The NDR obtains the DM data from 84 regions of the Russian Federation (15).

All of the patients have signed the informed consent form to provide their medical data for reporting to the register. The study protocol, based on the analysis of the depersonalized NDR dataset, was approved by the local ethics committee of Endocrinology Research Centre, Moscow, Russia, on April 30, 2020 (protocol №6).

Since March 20, 2020, three additional fields have been added for registration to the NDR:

- Diagnosed case of COVID-19 or viral pneumonia: Yes/No
- Laboratory or CT-confirmed COVID-19: Yes/No/Not done
- COVID-19/pneumonia outcome: Recovery/Death

As of November 25, 2021, the NDR included in total 4,919,826 patients with DM (i.e., 3.37% of the population of the Russian Federation); among them, 269,500 had T1DM, 4.55 million had T2DM, and 105,100 had other types of diabetes.

In total, the analysis covered 235,248 NDR records of patients with diabetes, who have had COVID-19 or viral pneumonia, with the specified outcome (death or recovery) in the period from March 20, 2020, to November 25, 2021. The data were reported to the NDR by local physicians based on the primary medical records, laboratory results, and the outcomes indicated in the certificates of the Russian State system of death and birth registration.

For 20 months of data reporting, 11,058 patients with T1DM and 224,190 with T2DM were available for the analysis. COVID-19 was confirmed by positive PCR testing in 186,364 patients (79.2%). The remaining 48,884 patients (20.8%) had no PCR tests performed or negative PCR; however, the COVID-19 diagnosis was confirmed by a CT scan. Those patients who had negative PCR testing but “positive” CT scans of the lungs were considered as highly suspected for COVID-19, so we assumed them to be eligible for inclusion in the study.

The dataset covered 35,088 cases of death and 200,160 cases of survivors. All of the included patients had clinical and laboratory data obtained at the last visit before COVID-19 during the period from March 20, 2020, to November 25, 2021.

**Figure 1:**
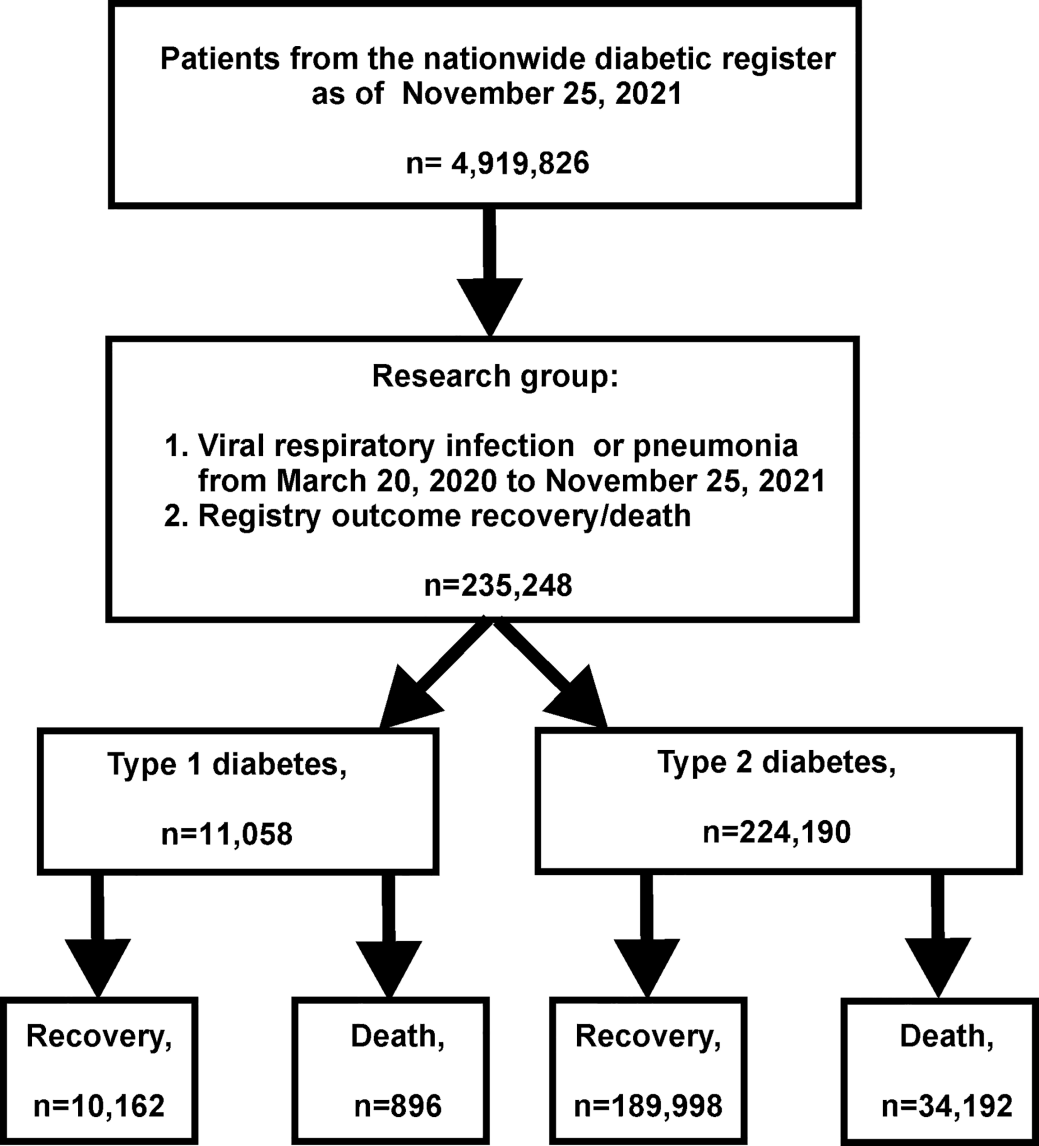
Study design.

The study design is presented in **Figure 1**.

### Objectives and outcomes

The objective of this analysis was to assess the association of demographic, clinical, and laboratory characteristics, pre-COVID-19 glucose-lowering therapy (in T2DM), and anti-COVID-19 vaccination status with the fatality cases due to COVID-19 and identify the risk factors for the death.

The CFR was calculated as the ratio of death cases (n, absolute number) to the total number of patients with COVID-19 (N, absolute number), presented in %.

### Covariates and factors

Covariates and factors were exported from the database of NDR. Age and DM duration were calculated for the index date as of November 25, 2021. The most recent laboratory data and measurements obtained at the last visit before COVID-19 were glycated hemoglobin (HbA1c) level, body mass index (BMI), systolic (SBP) and diastolic blood pressure (DBP), estimated glomerular filtration rate (eGFR), total cholesterol, high-density lipoprotein (HDL), low-density lipoprotein (LDL), and triglyceride (TG) levels, as well as qualitative factors: sex, history of confirmed atherosclerotic cardiovascular disease (ASCVD) and chronic kidney disease (CKD), anti-COVID-19 vaccination status in both DM types and in T2DM—the pre-COVID-19 glucose-lowering therapy [insulin, metformin, sulfonylureas (SU), inhibitors of dipeptidyl peptidase 4 (DPP-4 inhibitors), SGLT2 inhibitors, glucagon-like peptide-1 receptor agonists (arGLP-1)]. Acarbose and thiazolidinediones were not included in the analysis due to very low frequency in the Russian Federation. Individual drug risks were assessed as part of combination therapy.

The HbA1c level was measured with standardized methods in accordance with the Diabetes Control and Complications Trial (DCCT) and National Glycohemoglobin Standardization Program (NGSP) standard.

ASCVD was determined as one or more of these conditions: myocardial infarction, stroke, chronic heart disease, chronic heart failure, or revascularization surgery. CKD was defined as morning urine albumin/creatinine ratio >3 mg/mmol or 30 mg/g or eGFR <60 ml/min/1.73 m^2^ due to the standard CKD-Chronic Kidney Disease Epidemiology Collaboration Formula (EPI) formula.

### Statistical analysis

Statistical analysis of the data was carried out in the software packages Statistica 13.0 (Tibco, USA), SPSS 26 (IBM, USA), and in the R environment (version 3.6.3). Descriptive statistics was presented by medians and first, third quartiles [Q1; Q3] for quantitative characteristics and relative frequencies for qualitative ones.

Comparison of two independent groups for quantitative data was performed using the Mann–Whitney test (U-test). The frequencies of qualitative features were compared with each other using the chi-square test (χ^2^) and Fisher criterion.

The association between covariates and factors and mortality risk was expressed as the unadjusted odds ratio (OR) and 95% confidence interval (CI). OR >1 represented an increase of mortality, and those with <1 represented a decrease. Such covariates as age, HbA1c, and BMI were presented as binary factors by splitting them by cutoff points: 65 years, 7%, and 30 kg/m^2^, respectively. At the next step, the multivariate logistic regression analysis was conducted to calculate the adjusted OR for all factors used in univariate analysis.

To identify the most vulnerable group at risk of lethal outcome due to COVID-19, we conducted the analysis for combinations of the most significant factors, which showed the significance in the univariate analysis. Risk factors (OR >1) were ranked by OR value (calculated by univariate analysis) in descending order and protective factors (OR <1) in ascending order. The protective factor vaccination with OR <1 to include in group risk analysis was recalculated as the absence of vaccination. So, we have chosen six combinations of the most important factors out of 57 possible factor combinations in T1DM and 4,083 combinations in T2DM.

The level of significance was assumed if p < 0.05. For multiple comparisons of indicators, the critical level of significance (Р_0_) of statistical results was recalculated with Bonferroni correction.

## Results

The study included 235,248 patients with T1DM and T2DM from 84 regions of the Russian Federation, including 76,220 (32%) men and 159,028 (68%) women.

The CFR due to COVID-19 in total of both T1DM and T2DM patients was 14.9% (35,088 out of 235,248); in patients with T1DM, 8.1% (896 out of 11,058), and in patients with T2DM, 15.3% (34,192 out of 224,190).

According to the outcome, the clinical aspects of T1DM and T2DM patients are presented in **Tables 1**–**3**, respectively.

**Table 1:** Clinical characteristics of T1DM patients according to the outcome (death/recovery) (n = 11,058).

Factor		Recovery		Death		p-value
		N	Descriptive statistics	N	Descriptive statistics	
**Sex**	**Men, %**	5,103	50.22%	500	55.80%	0.001^2^
	**Women, %**	5,059	49.78%	396	44.20%	
**Diabetes duration, years, median [Q1; Q3]**		10,078	14 [7; 23]	896	21 [13; 31]	<0.001^1^
**Age, years, median [Q1; Q3]**		10,162	40 [31; 52]	896	55 [41; 65]	<0.001^1^
**HbA1c, %, median [Q1; Q3]**		6,195	7.7 [6.9; 8.8]	229	7.8 [7.0; 8.8]	0.277^1^
**BMI, kg/m^2^, median [Q1; Q3]**		7,648	24.75 [21.89; 28.09]	341	25.69 [22.68; 30.25]	<0.001^1^
**DBP, mmHg, median [Q1; Q3]**		6,379	80 [70; 80]	301	80 [78; 80]	<0.001^1^
**SBP, mmHg, median [Q1; Q3]**		6,389	120 [120; 130]	302	130 [120; 135]	<0.001^1^
**eGFR, ml/min/1.73 m^2^, median [Q1; Q3]**		5,905	92 [74; 109]	244	73 [43; 95]	<0.001^1^
**Total cholesterol, mmol/l, median [Q1; Q3]**		5,456	4.7 [4.1; 5.5]	213	4.7 [4.0; 5.5]	0.138^1^
**LDL, mmol/l, median [Q1; Q3]**		2,232	2.6 [2.1; 3.4]	75	2.7 [2.0; 3.4]	0.839^1^
**HDL, mmol/l, median [Q1; Q3]**		1,869	1.4 [1.2; 1.8]	61	1.2 [1.1; 1.5]	0.013^1^
**TG, mmol/l, median [Q1; Q3]**		2,691	1.1 [0.8; 1.6]	85	1.3 [1.1; 2.0]	<0.001^1^
**ASCVD, %**		2,913	28.67%	361	40.29%	<0.001^2^
**CKD, %**		4,316	42.47%	502	56.03%	<0.001^2^
**Anti-COVID-19**		746	40.13%	4	4.71%	<0.001^3^
**Vaccination, %**						
**Anti-COVID-19**	**Sputnik V (Gam-COVID-Vac), %**	581	5.72%	2	0.22%	–
**Vaccine**	**CoviVac, %**	19	0.19%	0	0.00%	
	**Sputnik Light, %**	54	0.53%	0	0.00%	
	**EpiVacCorona, %**	32	0.31%	0	0.00%	

^1^U-test.

^2^χ^2^.

^3^χ^2^ with Yates’s correction.

P_0_ = 0.05/16 = 0.003.

Data are % or median and first, third quartiles [Q1; Q3].

HbA1c, glycated hemoglobin level; BMI, body mass index; SBP, systolic blood pressure; DBP, diastolic blood pressure; HDL, high-density lipoprotein; LDL, low-density lipoprotein; TG, triglyceride; ASCVD, atherosclerotic cardiovascular disease; CKD, chronic kidney disease; eGFR, estimated glomerular filtration rate; COVID19, coronavirus disease.

**Table 2:** Clinical characteristics of T2DM patients according to the clinical outcome (n = 224,190).

Factor		Recovery		Death		p-value
		N	Descriptive statistics	N	Descriptive statistics	
**Sex**	**Men, %**	58,793	30.94%	11,824	34.58%	<0.001^2^
	**Women, %**	131,205	69.06%	22,368	65.42%	
**Diabetes duration, years, median [Q1; Q3]**		186,411	7 [2; 12]	34,192	10 [6; 15]	<0.001^1^
**Age, years, median [Q1; Q3]**		189,998	65 [59; 71]	34,192	72 [65; 79]	<0.001^1^
**HbA1c, %, median [Q1; Q3]**		97,702	7.2 [6.6; 8.1]	6,963	7.3 [6.8; 8.2]	<0.001^1^
**BMI, kg/m^2^, median [Q1; Q3]**		137,752	31.89 [28.52; 35.84]	10,794	32.03 [28.40; 36.44]	<0.001^1^
**DBP, mmHg, median [Q1; Q3]**		119,222	80 [80; 85]	9,348	80 [80; 90]	<0.001^1^
**SBP, mmHg, median [Q1; Q3]**		119,310	130 [130; 140]	9,355	130 [130; 140]	<0.001^1^
**eGFR, ml/min/1.73 m^2^, median [Q1; Q3]**		108,347	72 [60; 87]	7,434	66 [52; 81]	<0.001^1^
**Total cholesterol, mmol/l, median [Q1; Q3]**		99,967	5.0 [4.2; 5.8]	7,040	5.0 [4.2; 5.6]	<0.001^1^
**LDL, mmol/l, median [Q1; Q3]**		36,715	2.7 [2.1; 3.5]	2,154	2.6 [2.0; 3.3]	<0.001^1^
**HDL, mmol/l, median [Q1; Q3]**		30,210	1.3 [1.0; 1.5]	1,817	1.2 [1.0; 1.6]	0.630^1^
**TG, mmol/l, median [Q1; Q3]**		42,684	1.6 [1.2; 2.2]	2,860	1.5 [1.1; 2.0]	<0.001^1^
**ASCVD, %**		109,478	57.62%	17,729	51.85%	<0.001^2^
**CKD, %**		69,475	36.57%	11,261	32.93%	<0.001^2^
**Anti-COVID-19**		13,278	39.50%	352	11.22%	<0.001^2^
**Vaccination, %**					
**Anti-СOVID-19 Vaccine**	**Sputnik V (Gam-COVID-Vac), %**	11,056	5.82%	253	0.74%	<0.001^3^
	**CoviVac, %**	84	0.04%	3	0.01%	
	**Sputnik Light, %**	782	0.41%	1	0.00%	
	**EpiVacCorona, %**	455	0.24%	18	0.05%	

^1^U-test.

^2^χ^2^.

^3^Fisher’s test.

P_0_ = 0.05/16 = 0.003.

Data are % or median and first, third quartiles [Q1; Q3].

HbA1c, glycated hemoglobin level; BMI, body mass index; SBP, systolic blood pressure; DBP, diastolic blood pressure; HDL, high-density lipoprotein; LDL, low-density lipoprotein; TG, triglyceride; ASCVD, atherosclerotic cardiovascular disease; CKD, chronic kidney disease.

**Table 3:** Comparison of CFR in T2DM groups according to the kind of receiving pre-COVID-19 glucose-lowering therapy (yes/no) (n = 224,190).

Factor	On therapy			No therapy			p-value
	Recovery	Death	CFR, %	Recovery	Death	CFR, %	
**Insulin**	47,112	11,159	19.2%	142,866	23,033	13.9%	<0.001^2^
**Metformin**	139,637	21,471	13.3%	50,361	12,721	20.2%	<0.001^2^
**SU**	76,548	16,248	17.5%	113,450	17,944	13.7%	<0.001^2^
**DPP-4 inhibitors**	25,508	2,860	10.1%	164,490	31,332	16.0%	<0.001^2^
**SGLT2 inhibitors**	15,728	1,364	8.0%	174,270	32,828	15.9%	<0.001^2^
**arGLP-1**	753	52	6.5%	189,245	34,140	15.3%	<0.001^2^

^2^χ^2^.

Data are %.

SU, sulfonylurea; DPP-4 inhibitors, inhibitors of dipeptidyl peptidase 4; SGLT2 inhibitors, sodium-glucose co-transporter-2 inhibitors; arGLP-1, glucagon-like peptide-1 receptor agonists.

People with both DM types who died, when compared with the recovered, were significantly older, and most of them were men and had a longer DM duration, higher BMI, higher SBP and DBP, and less eGFR (**Tables 1**, **2**). Additionally, in T1DM, there were significantly higher TG levels (**Table 1**); in T2DM, higher HbA1c levels (**Table 2**).

Patients with T1DM who died were more likely to have a history of ASCVD and CKD (**Table 1**). Surprisingly and in contrast to T1DM, in recovered compared with the T2DM patients who died, there were more ASCVD and CKD and higher TG and LDL levels (**Table 2**).

**Figure 2:**
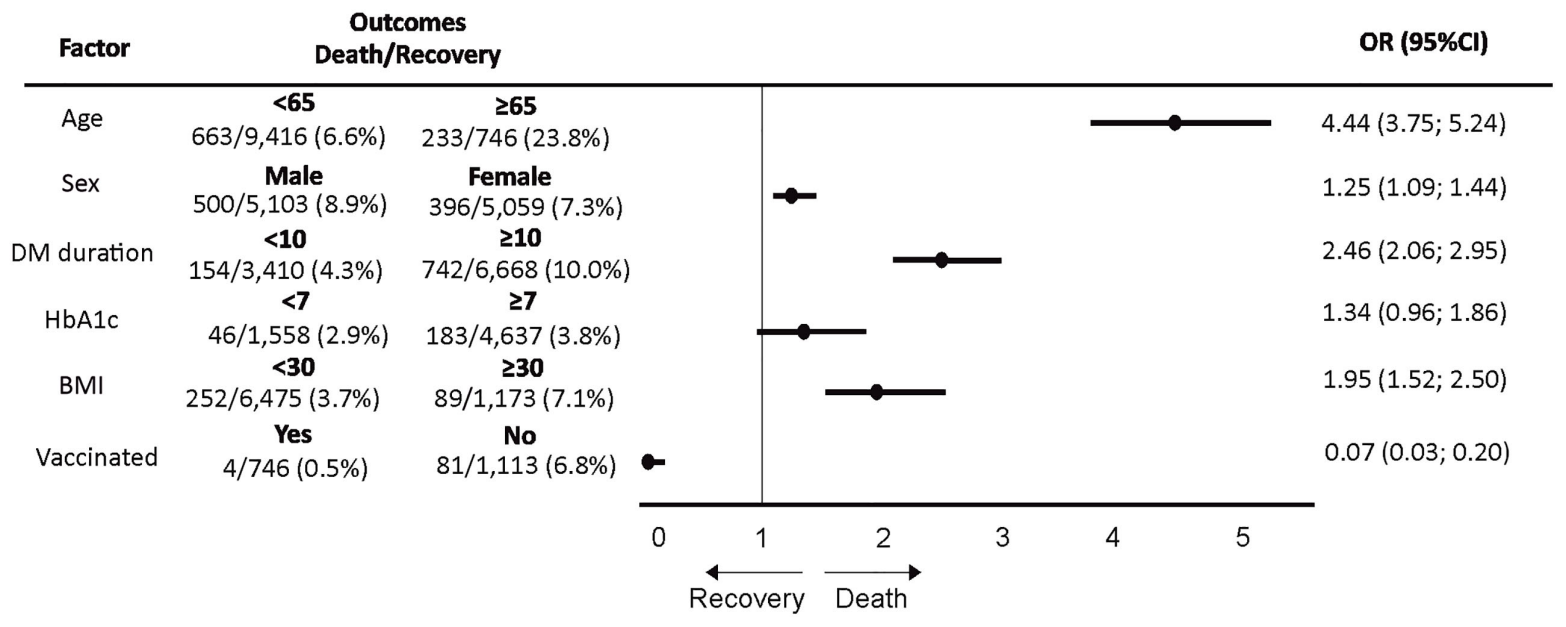
The analysis for association [odds ratio (OR)] between demographic and clinical characteristics and coronavirus disease 2019 (COVID-19) case fatality rate (recovery or death outcomes) in patients with type 1 diabetes mellitus (T1DM).

**Figure 3:**
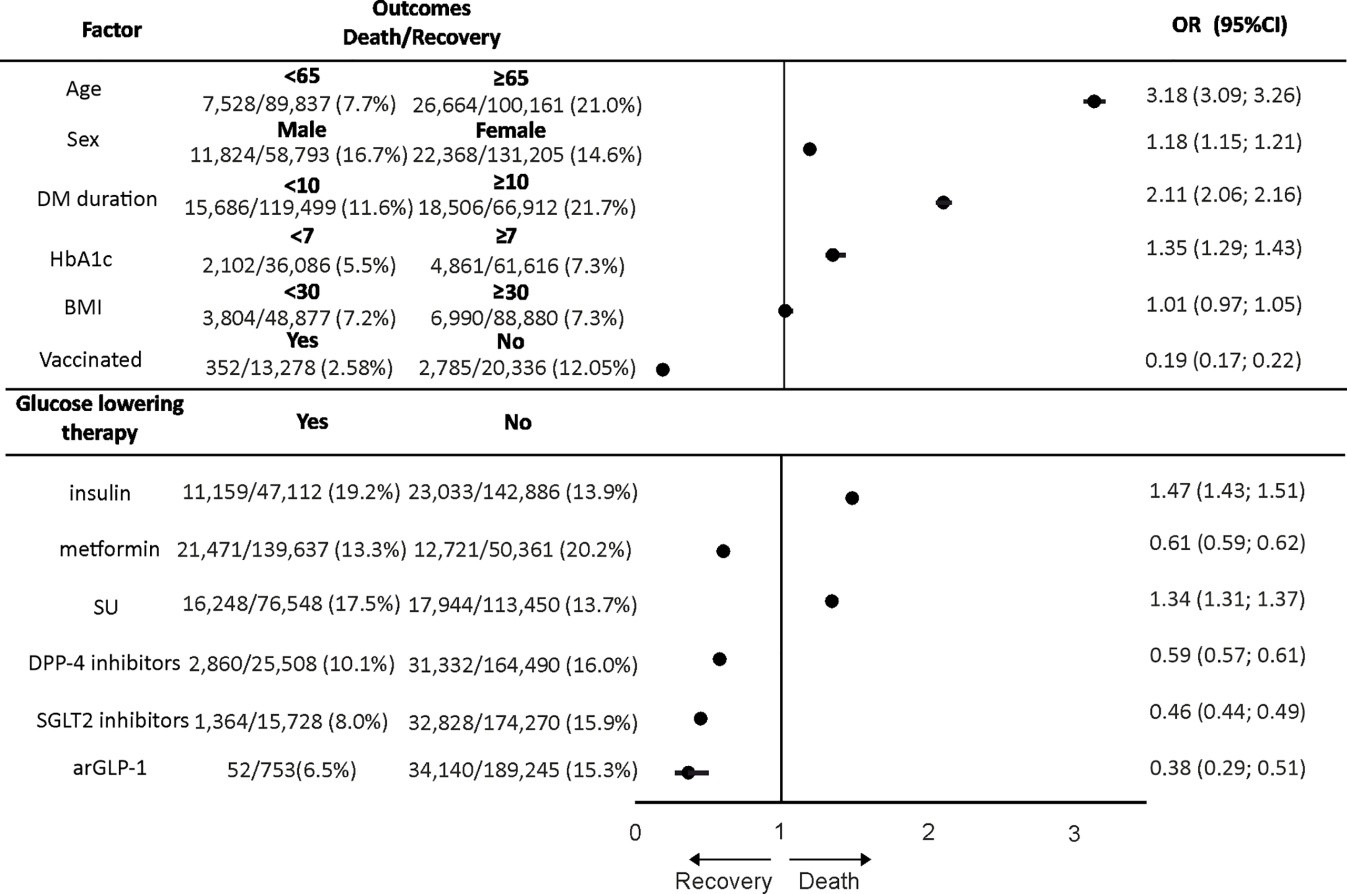
The analysis for association [odds ratio (OR)] between demographic and clinical characteristics, glucose-lowering therapy, and coronavirus disease 2019 (COVID-19) case fatality rate (recovery or death outcomes) in patients with type 2 diabetes mellitus (T2DM).

Univariate analysis (crude OR) for COVID-19 outcome risk factors showed that increased CFR was associated with the male population [OR = 1.25 (95% CI: 1.09–1.44) in T1DM and 1.18 (95% CI: 1.15–1.21) in T2DM], age ≥65 years [OR = 4.44 (95% CI: 3.75–5.24) in T1DM and 3.18 (95% CI: 3.09–3.26) in T2DM], DM duration ≥10 years [OR = 2.46 (95% CI: 2.06–2.95) in T1DM and 2.11 (95% CI: 2.06–2.16) in T2DM], BMI ≥30 kg/m^2^ [OR = 1.95 (95% CI: 1.52–2.50) in T1DM], and HbA1c ≥7% [OR = 1.35 (95% CI: 1.29–1.43) in T2DM] (**Figures 2**, **3**).

The CFR was significantly higher in T2DM patients receiving insulin and SU and significantly lower in those taking metformin, DPP-4 inhibitors, SGLT2 inhibitors, and arGLP-1 (on therapy vs. no therapy) before the viral disease (**Table 3**).

Pre-COVID-19 glucose-lowering therapy in T2DM was differently associated with death outcomes (OR): 0.61 (95% CI: 0.59–0.62) for metformin, 0.59 (95% CI: 0.57–0.61) for DPP-4 inhibitors, 0.46 (95% CI: 0.44–0.49) for SGLT2 inhibitors, 0.38 (95% CI: 0.29–0.51) for arGLP-1, 1.34 (95% CI: 1.31–1.37) for SU, and 1.47 (95% CI: 1.43–1.51) for insulin therapy (**Figure 3**).

Patients with T1DM and T2DM who were vaccinated against COVID-19 had likewise less CFR (**Tables 1**, **2**). *A posteriori* comparison of fatality rate in groups of T2DM patients who were vaccinated by different vaccines showed that the CFR was significantly lower in the group with Sputnik Light vaccine than that in the group with Gam-COVID-Vac, CoviVac, and EpiVacCorona vaccines (p = 0.002, p = 0.008, p < 0.001, χ^2^ with Yates’s correction, respectively). Anti-COVID-19 vaccination was associated with lower CFR in both DM types [0.07 (95% CI: 0.03–0.20) in T1DM and 0.19 (95% CI: 0.17–0.22) in T2DM] (**Figures 2**, **3**).

At the next step, we have provided multivariate analysis to calculate the adjusted OR. It should be noticed that most factors lost statistical significance according to the p-value due to multivariate analysis (**Tables S1**, **S2**).

The combinations of the most important factors were formed according to the algorithm based on the univariate analysis. The following six factor combinations (three for T1DM and three for T2DM) were chosen:

For T1DM:

1. Vaccine absence, age ≥65 years, DM duration ≥10 years
2. Vaccine absence, age ≥65 years, DM duration ≥10 years, BMI ≥30 kg/m^2^
3. Vaccine absence, age ≥65 years, DM duration ≥10 years, male sex

For T2DM:

1. Vaccine absence, age ≥65 years, DM duration ≥10 years
2. Vaccine absence, age ≥65 years, DM duration ≥10 years, insulin
3. Vaccine absence, age ≥65 years, DM duration ≥10 years, HbA1c ≥7%

The results of combination analysis are presented in **Tables S3**, **S4**. According to our results, there is a substantial increase in the risk of lethal outcome in the presence of factor combination compared to the risk of individual factors. For T1DM, the most important combination is vaccine absence + age ≥65 years + DM duration ≥10 years that increased the risk of lethal outcome compared to the individual risk of age and DM duration in 2–385 and 3.5–685 times, respectively (based on boards of ORs and CIs; CIs do not intersect), while the high significance of the vaccine is comparable both individually and in combination. For T2DM, the most important combination is vaccine absence + age ≥65 years + DM duration ≥10 years + insulin that increased the risk of lethal outcome compared to the individual risk of these factors in 5–7, 7–13, 11–19, and 16–28 times, respectively (based on boards of ORs and CIs; CIs do not intersect).

## Discussion

According to epidemiological studies, DM is one of the most common comorbidities in COVID-19. About 30%–40% of severe COVID-19 cases occurred in people with T2DM or T1DM (16).

The systematic review and meta−analysis of 87 studies published by Corona et al. (17) showed that, among the associated morbidities, DM was the strongest predictor of in-hospital mortality; it was 25% in the United States, 20% in Europe, and 13% in Asia. But only a few publications represent the analyses of the mortality or CFR in people with T1DM and T2DM based on the population-based nationwide observational studies using the data from NDRs (8, 9, 18).

Our study was based on the analysis of the nationwide DM registry population. According to our data, CFR was 14.9% in the total group of DM patients, 8.1% in T1DM, and 15.3% in T2DM. In the general population of the Russian Federation as of November 2021, the reported COVID-19-related CFR was around 3% (19). Thus, the CFR in diabetes was 2.7 times higher in T1DM and 5.1 times higher in T2DM than that in the general population.

The similar increase in mortality due to COVID-19 in people with both DM types was demonstrated in the population-based study conducted in England (9). In this study, the increased COVID-19-related mortality was also associated with the male population, older age, and poor glycemic control as in our study. Additionally, it was found that previous stroke, heart failure, and CKD were associated with increased COVID-19-related mortality in both T1DM and T2DM (9). In our study, the higher level of fatal outcomes in people with ASCVD and CKD was found only in T1DM but not in T2DM. In T1DM with ASCVD and CKD, the CFR was higher than that in T1DM without these comorbidities (11.0% vs. 6.9%, p < 0.001 for ASCVD; 10.4% vs. 6.3%, p < 0.001 for CKD).

In T2DM in our study, the frequencies of ASCVD and CKD were unexpectedly higher in the recovered group than those in the group with lethal outcome. We suppose that people with T2DM and comorbidities, unlike those without, were additionally taking the organoprotective medications for blood pressure lowering (renin-angiotensin system blockers), statins and antiplatelet therapy. Such therapy is mostly associated with a lower mortality risk due to COVID-19 (20–22). Unfortunately, in our study, we did not analyze the association of taking these medications and COVID-19 outcome due to the absence of data.

The association between the level of HbA1c and COVID-19 mortality rate in patients with DM is still contradictory. A systematic review and meta‐analysis published by Prattichizzo et al. (23) showed that a higher HbA1c was associated with an increased COVID‐19-related mortality or worsening of symptoms in patients with DM [OR = 1.01 (95% CI: 1.01–1.01), p < 0.00001]. This analysis included reporting data regarding HbA1c values before or during hospital admission and involved 1,524,573 DM patients with available HbA1c data, 298,850 of them being with T1DM. The authors mention that the findings must be interpreted with caution for a variety of reasons: the large heterogeneity in study designs, patients’ characteristics, and methods of descriptive statistics. In our study, HbA1c ≥7% was significantly associated with CFR only in T2DM [OR = 1.35 (95% CI: 1.29–1.43), p < 0.001] but not in T1DM [OR = 1.34 (95% CI: 0.96–1.86), p = 0.083]. It should be mentioned that HbA1c obtained outside of hospitals and not just before the infection, but at the last outpatient visit before the infection, might impact the observed results.

Obesity is a recognized risk factor for COVID-19 severity (hospitalization, intensive care unit admission, the need for invasive ventilation) but is not so strongly associated with COVID-19 mortality (24, 25). Some studies found a nonlinear J-curve association between BMI and COVID-19 severity and mortality (26). In our study, obesity (BMI ≥30 kg/m^2^) was associated with an increased risk of fatal outcomes [OR = 1.95 (95% CI: 1.52–2.50), p < 0.001] in T1DM and there was no significant association in T2DM [OR = 1.01 (95% CI: 0.97–1.05), p = 0.618]. Our data for T2DM were obtained on a rather large sample size (n = 224,190) that elevates the study’s statistical power and allows to assume that although obesity is associated with more severe COVID-19 and disease progression, paradoxically, these associations do not strongly appear to result in an increased risk of death.

An important aim of our study was to investigate whether there are associations between prescription of different classes of antidiabetic drugs and the risk of COVID-19-related fatal outcomes in T2DM patients. In our nationwide observational cohort study, based on the large register database, there was statistical evidence that the use of metformin, DPP-4 inhibitors, SGLT2 inhibitors, and arGLP-1 in people with T2DM before COVID-19 infection is associated with a lower fatality rate compared to those who did not use these drugs before COVID-19. Quite the opposite, those people who were on insulin or SU therapy were at a higher risk for death than those who did not take these drugs before the infection.

Up to now, the largest nationwide observational study to examine the associations of prescribed antidiabetic drugs with COVID-19-related mortality is provided by Khunti et al. (12) in England. In that study, the COVID-19-related mortality in T2DM was significantly lower in patients prescribed metformin, SGLT2 inhibitors, and SU and higher in patients prescribed DPP-4 inhibitors and insulin vs. those not prescribed these medications (12).

There is no controversy in the opinions of previously published studies about the association of the decreased COVID-19 mortality with metformin. Three meta-analyses demonstrated that metformin was associated with lower COVID-19 mortality: OR = 0.66 (95% CI: 0.56–0.78) (27), OR = 0.64 (95% CI: 0.43–0.97) (28), OR = 0.37 (95% CI: 0.16–0.59) (29). In all studies, demonstrating the protective role of metformin, the drug was used before developing an acute viral infection, respiratory distress syndrome, and severe hypoxia. Some hypotheses can explain the protective effect of metformin on COVID-19 outcomes. Metformin may cause phosphorylation of angiotensin-converting enzyme cellular receptor for SARS-CoV-2 that may cause the protective properties of this drug in coronavirus infection. As a result, the receptor’s conformation could be changed, causing a decrease in its binding with the coronavirus (30). Speculatively, we can suggest that metformin may play its protective role by activating various cellular mechanisms, lowering vitamin B12 levels and immunosuppression, inhibiting the Phosphatidylinositol 3-kinase, alpha kinase threonine-protein, mammalian target of rapamycin (PI3K/AKT/mTOR) pathway, reducing the formation of blood clots, reducing lung damage and the severity of fibrosis, interrupting endocytosis due to a decrease in the acidity of endosomes and lysosomes, and reducing the synthesis of inflammatory cytokines such as Interleukin-6 (IL-6) and tumor necrosis factor α (TNF-α) (30).

There are much less data on the impact of DPP-4 inhibitors on the outcomes of COVID-19, and the available information is contradictory (31). In our study, therapy with DPP-4 inhibitors prior to virus infection was associated with a lower risk of death [OR = 0.59 (95% CI: 0.57–0.61), p < 0.001]. The same conclusion was made in the meta-analysis by Yang et al. (32) who analyzed 4 studies with a total of 1,933 patients with COVID-19 and T2DM. According to this meta-analysis, the use of DPP-4 inhibitors was negatively associated with the risk of mortality [OR = 0.58 (95% CI: 0.34–0.99)]. Another meta-analysis included 4,477 T2DM patients from 9 studies also confirmed that DPP-4 inhibitor use was associated with lower mortality in COVID-19 patients (33). The safety of DPP-4 inhibitors was confirmed in a systematic review by Kan et al. (29), which revealed no significant difference in mortality between DPP-4 inhibitor users and non-users. Unlike these studies, Khunti et al. (12) showed a higher mortality risk for T2DM patients on DPP-4 inhibitor therapy. We suppose that such differences in the results may be related to dissimilarities in sample size or confounding adjustment.

There is some encouraging information that SGLT2 inhibitors can protect against a severe course of COVID-19 and poor outcome (12, 34). DARE-19 is the first randomized study that aimed to investigate the effect of SGLT2 inhibitors on the rate of poor outcomes in T2DM patients hospitalized with COVID-19 (35). The frequency of cardiovascular, renal, and/or respiratory complications, mortality from all causes, and parameters of clinical recovery (e.g., length of hospitalization) were assessed in DARE-19. The study included 1,200 patients randomized to receive dapagliflozin or placebo. In patients with cardiometabolic risk factors hospitalized with COVID-19, dapagliflozin did not reduce the risk of organ dysfunction or death significantly. Nevertheless, this study demonstrated a high safety profile when using SGLT2 inhibitors even in COVID-19 patients with multiple risk factors (34). The results of our study demonstrated fewer fatal outcomes in T2DM patients prescribed SGLT2 inhibitors prior to infection [OR = 0.46 (95% CI: 0.44–0.49), p < 0.001]. The potential protective effect of SGLT2 inhibitors in COVID-19 may be associated with multiple pleiotropic properties, including anti-inflammatory action and a decrease in the severity of oxidative stress, improved myocardial and endothelial function, improved oxygen delivery, and increased urine output (36) and with a well-known cardio- and nephroprotective effect of this class of drugs (37).

There is still lack of information about arGLP-1 and their association with COVID-19 outcomes. A recently published meta-analysis that included 9 studies with 19,660 T2DM patients infected with COVID-19 (38) suggests that preadmission use of arGLP-1 may offer beneficial effects on COVID-19 mortality in patients with DM. The study by Khunti et al. (12) and our study demonstrated the same positive results. The beneficial effect of arGLP-1 may be associated with their positive cardiovascular effects in T2DM patients both with established cardiovascular diseases and those at high risk of cardiovascular diseases (39).

Plenty of publications demonstrated a higher mortality rate in T2DM patients receiving insulin before an acute COVID-19 (12, 29, 40, 41). Khunti et al. (12) found that insulin users had increased risk of COVID-19-related mortality [hazard ratio (HR) = 1.42 (95% CI: 1.35–1.49)]. A meta-analysis made by Kan et al. (29) showed a higher mortality risk in insulin users (pooled OR = 2.20; p = 0.002). Chen et al. (40) demonstrated a higher level of fatal outcomes of COVID-19 in patients with T2DM using insulin (22.5% vs. 6.1%, p = 0.021). A meta-analysis by Yang et al. (41) demonstrated an increased risk of COVID-19 mortality in T2DM patients using insulin [OR = 2.10 (95% CI: 1.51–2.93)]. This meta-analysis showed that T2DM patients on insulin therapy were much more comorbid than those without insulin therapy. The frequencies of ASCVD, high BP, and CKD were 68%, 52%, and 45% in insulin users vs. 57%, 46%, and 29% in insulin non-users (p < 0.001, respectively) (41). The findings of our study confirm the higher rate of fatal outcomes in T2DM patients who were treated with insulin before they were infected with COVID-19 [OR = 1.47 (95% CI: 1.43–1.51), p < 0.001]. We suggest that interpretation of such results should be done with caution. The higher fatality rate in insulin-treated T2DM patients associated not with insulin *per se*, and the data of T1DM patients, all of whom are taking insulin, are the great evidence. That might refer to the poorer glycemic control of such patients, longer DM duration, and more severity due to comorbidity state (cardiovascular and renal). In our study, we did not make the subanalysis of the reasons for poor clinical outcomes in people with T2DM taking insulin. That is our plan for future analysis.

One of the most popular classes of antidiabetic drugs in T2DM is SU. In the study by Khunti et al. (12), SU intake lowered the risk of COVID-19-related mortality [HR = 0.94 (95% CI: 0.89–0.99)]. The same conclusion was made based on the meta-analysis by Kan (29) —lower mortality in SU users [OR = 0.80 (95% CI: 0.66–0.96)]. In a systematic review made by Han et al. (42), prescribing of SU/glinides slightly decreased the mortality [pooled OR = 0.93 (95% CI: 0.89–0.98), p = 0.004] but did not prevent poor composite outcomes such as ICU admission, respiratory distress syndrome, and invasive ventilation, [pooled OR = 1.48 (95% CI: 0.61–3.60), p = 0.384]. In our study, we received quite the opposite result concerning SU uses. The analysis of our database showed that SU therapy in T2DM was associated with a higher fatality risk than that without SU [OR = 1.34 (95% CI: 1.31–1.37), p < 0.001]. The reason for such discrepancy is not so clear. In the Russian Federation, the frequency of SU prescribing in usual clinical practice decreased from 2016 to 2020 from 56% to 47%; however, it remains rather high (43). About 27% of T2DM patients receive SU drugs as monotherapy (43). Previously published data demonstrated that SU monotherapy vs. metformin monotherapy or switching to SU after metformin was associated with an increased risk of cardiovascular events and all-cause mortality (44–46). We suggest that the negative effect of SU therapy on COVID-19 CFR in the Russian population may be associated with a rather high proportion of people on SU monotherapy.

In the Russian Federation, four anti-COVID-19 vaccines are approved for preventing the new coronavirus disease. They are Sputnik V (Gam-COVID-Vac), Sputnik Light, CoviVac, and EpiVacCorona. The results of our analysis showed that anti-COVID-19 vaccination was associated with a lower risk for CFR in both DM types: OR = 0.07 (95% CI: 0.03–0.20) in T1DM and 0.19 (95% CI: 0.17–0.22) in T2DM. Noteworthy, in the presumably non-diabetic population, Gam-COVID-Vac usage OR was estimated to be 0.08 (95% CI: 0.04–0.14) (47). A similar result was revealed in a study by Dispinseri et al. (48). The protective effect of neutralizing antibodies against SARS-CoV-2 was confirmed in the general population including DM patients [HR = 0.28 (95% CI: 0.08–0.98), p = 0.046]. However, it was not indicated whether the antibodies were the result of vaccination or a previous COVID-19. The Office for National Statistics in England reported that the age-standardized mortality rate for deaths involving COVID-19 is 32 times higher for unvaccinated people than those who received the second dose (849.7 cases vs. 26.2 per 100,000 person-years, respectively) (49).

In an attempt to verify the most important predictors of lethal outcome, we performed a multivariate analysis. Multivariate regression analysis failed to reveal an increase in the significance of any certain factors. Then, the ranking of factors was performed based on OR values, and the combinations of factors with the highest OR values were selected. Groups’ factor analysis allowed us to identify the categories of patients most at risk of lethal outcome, including age ≥65 years, DM duration of diabetes ≥10 years, and the absence of a vaccine as the most important risk factors for both DM types, indicating an additive negative effect of factors’ combination. According to our results, in the presence of several factors, the risk of lethal outcome increases several times compared to the risk in the presence of individual factors. So, we could conclude that this group required the most careful monitoring in case of COVID-19.

### Study limitations

The research was based on the retrospective analysis of the nationwide diabetes register cohort. The research included patients with viral respiratory infection or pneumonia according to the physicians’ reports to the NDR. COVID-19 was laboratory-confirmed by a positive PCR test in 186,364 patients and confirmed by CT scan in 48,884 patients, so not all patients had the laboratory-confirmed disease.

The reported patients with COVID-19 were treated differently either at home or in hospitals, with doctors having different experiences in treatment approach and drug supply that might influence the treatment outcomes. The study focused on pre-COVID-19 therapy prescription and did not consider potential changes or discontinuation of treatment, particularly in COVID-19 hospital settings.

Another important limitation of this study was the inability to use non-diabetic risk factors that can affect the outcome of death in COVID-19, such as non-diabetic treatment and immunodeficiency, since they were not recorded in the diabetes registry.

We analyzed drug administration in monotherapy and in the presence of combined drugs. Individual drug risks were assessed as part of combination therapy.

## Conclusion

Retrospective analysis of fatal outcomes in 235,248 DM patients with COVID-19 revealed a set of risk factors associated with death outcome. Both in T1DM and T2DM, the risk factors were the male population, age 65 years or above, and DM duration of 10 years or above; additionally, in T2DM, HbA1c ≥7%; in T1DM, BMI ≥30 kg/m^2^. Therapy with metformin, DPP-4 inhibitors, SGLT2 inhibitors, arGLP-1 before the infection and anti-COVID-19 vaccination were associated with a lower CFR, while SU and insulin therapy was associated with a higher CFR due to COVID-19. The group with the highest risk of lethal outcome includes patients with a combination of factors such as an age ≥65 years, DM duration ≥10 years, and the absence of a vaccine in both DM types that required the most careful monitoring in case of COVID-19.

## Data availability statement

The datasets presented in this article are not readily available because according to local ethical policy data are available on request from the author upon the permission of local ethical committee of Endocrinology Research Centre, Moscow, Russia. Requests to access the datasets should be directed to corresponding author.

## Ethics statement

The studies involving human participants (human data of register medical reports) were reviewed and approved by local ethics committee of Endocrinology Research Centre, Moscow, Russia. The patients/participants provided their written informed consent for use of medical data register records for the study.

## Author contributions

MS, OV, ID and NM conceived and designed the study. AE, AD, and OV analyzed the data. OV, AD, AE, MS wrote the paper. ID and NM have done final revision of article. All authors, reviewed, edited, and approved the manuscript.

## Funding

The study was supported by Ministry of Science and Higher Education of the Russian Federation (agreement no. 075-15-2022-310).

## Conflict of interest

The authors declare that the research was conducted in the absence of any commercial or financial relationships that could be construed as a potential conflict of interest.

## Publisher’s note

All claims expressed in this article are solely those of the authors and do not necessarily represent those of their affiliated organizations, or those of the publisher, the editors and the reviewers. Any product that may be evaluated in this article, or claim that may be made by its manufacturer, is not guaranteed or endorsed by the publisher.
