# Supplementary material for: Risk factors for COVID-19 case fatality rate in people with type 1 and type 2 diabetes mellitus: A nationwide retrospective cohort study of 235,248 patients in the Russian Federation
Source: Front Endocrinol (Lausanne). 2022 Aug 9;13:909874. doi: 10.3389/fendo.2022.909874 (PMC9396282; doi:10.3389/fendo.2022.909874)
Supplement: Supplementary file 1 [file Table_1.docx]

Q2 Please provide new Supplementary Tables with the changes you've mentioned.

**Table S1. The multivariate analysis for association (adjusted odds ratio, OR) between demographic and clinical characteristics with lethal outcome due to COVID-19 in patients with T1DM (n=11,058)**

| **Factor** | **Adjusted OR (95% CI)** | **p-value** |
| --- | --- | --- |
| Age ≥65 years | 7.516 (2.785; 20.283) | **<0.001** |
| Male sex | 1.706 (0.702, 4.143) | 0.238 |
| Duration DM ≥10 years | 2.110 (0.579; 7.685) | 0.258 |
| HbA1c ≥7% | 1.920 (0.550; 6.703) | 0.306 |
| BMI ≥30 kg/m^2^ | 1.148 (0.393; 3.353) | 0.800 |
| Vaccinated | 0.181 (0.052; 0.628) | **0.007** |

HbA1c – glycated haemoglobin level, BMI – body mass index

DM – diabetes mellitus.

**Table S2. The multivariate analysis for association (adjusted odds ratio, OR) between demographic, clinical characteristics and glucose-lowering therapy with lethal outcome due to COVID-19 in patients with T2DM (n=224,190**)

| **Factor** | **Adjusted OR (95% CI)** | **p-value** |
| --- | --- | --- |
| Age ≥65 years | 2.289 (1.892; 2.769) | <0.001 |
| Male sex | 1.410 (1.182, 1.682) | <0.001 |
| Duration DM ≥10 years | 1.924 (1.606; 2.305) | <0.001 |
| HbA1c ≥7% | 1.187 (0.999; 1.411) | 0.052 |
| BMI ≥30 kg/m^2^ | 0.948 (0.790; 1.138) | 0.568 |
| Vaccinated | 0.314 (0.256; 0.385) | <0.001 |
| Insulin | 1.309 (1.071; 1.601) | 0.009 |
| Metformin | 0.731 (0.608; 0.877) | 0.001 |
| Sulfonamide | 1.256 (1.058; 1.491) | 0.009 |
| DPP-4 inhibitors | 0.715 (0.553; 0.923) | 0.010 |
| SGLT2 inhibitors | 0.751 (0.562; 1.003) | 0.052 |
| arGLP-1 | 0.731 (0.177; 3.012) | 0.665 |

HbA1c – glycated haemoglobin level, BMI – body mass index, SU – sulfonylurea, DPP-4 inhibitors – inhibitors of dipeptidyl peptidase 4, SGLT2 inhibitors – sodium-glucose co-transporter-2 inhibitors, arGLP-1 – glucagon-like peptide-1 receptor agonists

DM – diabetes mellitus.

**Table S3. The analysis for association (odds ratio, OR) of combinations with lethal outcome due to COVID-19 in patients with T1DM (n=11,058)**

| **Factor** | **OR (95% CI)** |
| --- | --- |
| Vaccine absence, age ≥65 years, DM duration ≥10 years | 120.57 (7.19-2020.70) |
| Vaccine absence, age ≥65 years, DM duration ≥10 years, BMI ≥30 kg/m^2^ | 53.54 (2.66-1078.13) |
| Vaccine absence, age ≥65 years, DM duration ≥10 years, male sex | 75.00 (4.28-1314.13) |

BMI – body mass index

DM – diabetes mellitus.

**Table S4. The analysis for association (odds ratio, OR) of factors` combinations with lethal outcome due to COVID-19 in patients with T2DM (n=224,190**)

| **Factor** | **OR (95% CI)** |
| --- | --- |
| Vaccine absence, age ≥65 years, DM duration ≥10 years | 26.20 (19.59-35.06) |
| Vaccine absence, age ≥65 years, DM duration ≥10 years, insulin | 30.23 (21.78-41.98) |
| Vaccine absence, age ≥65 years, DM duration ≥10 years, HbA1c ≥7% | 22.22 (9.83-50.23) |

HbA1c – glycated haemoglobin level

DM – diabetes mellitus.
